# Supplementary material for: Topic-driven toxicity: Exploring the relationship between online toxicity and news topics
Source: PLoS One. 2020 Feb 21;15(2):e0228723. doi: 10.1371/journal.pone.0228723 (PMC7034861; doi:10.1371/journal.pone.0228723)
Supplement: S1 Table — Note: “religion” was discarded from the analysis because the class contained only 3 videos. (DOCX) [file pone.0228723.s003.docx]

**Grouping of data into superclasses. Note: “religion” was discarded from the analysis because the class contained only 3 videos.**

| **superclass** | **topic** |  | **superclass** | **topic** |
| --- | --- | --- | --- | --- |
| africa | africa |  | middle east | iraq |
|  | libya |  |  | egypt |
|  | kenya |  |  | middle east |
|  | democratic republic of congo |  |  | iran |
| arts & culture | arts & culture |  |  | afghanistan |
| asia | asia pacific |  |  | gcc |
|  | asia |  |  | lebanon |
|  | india |  |  | qatar |
|  | turkey |  |  | jordan |
|  | myanmar |  |  | saudi arabia |
|  | pakistan |  |  | yemen |
|  | china |  |  | kurds |
|  | north korea |  | politics | politics |
|  | japan |  |  | elections |
|  | bangladesh |  |  | corruption |
|  | south korea |  |  | protests |
|  | philippines |  |  | un |
| business & economy | business & economy |  |  | gun violence |
| environment & weather | environment |  |  | women’s rights |
|  | weather |  |  | immigration |
|  | climate sos |  |  | education |
| europe | europe |  | racism | racism |
|  | united kingdom |  | religion | islam |
|  | france |  |  | religion |
|  | catalonia |  | russia | russia |
|  | germany |  | science & technology | science & technology |
| health | health |  | sport | sport |
| human rights | human rights |  |  | football |
| israel-palestine | palestine |  | us & canada | us & canada |
|  | israel |  |  | united states |
|  | israeli–palestinian conflict |  |  | donald trump |
|  | gaza |  |  | canada |
|  | jerusalem |  | war & conflict | war & conflict |
| latin america | latin america |  |  | syria’s civil war |
|  | mexico |  |  | battle for mosul |
| media | media |  |  | refugees |
|  | facebook |  |  | isis |
|  |  |  |  | humanitarian crises |
