# Supplementary material for: Topic-driven toxicity: Exploring the relationship between online toxicity and news topics
Source: PLoS One. 2020 Feb 21;15(2):e0228723. doi: 10.1371/journal.pone.0228723 (PMC7034861; doi:10.1371/journal.pone.0228723)
Supplement: S2 Table — (DOCX) [file pone.0228723.s004.docx]

**Summary of statistical test results**

| *Estimation:* | | *Heteroskedasticity correction:* | | *no* | *yes* | *no* | *yes* | *yes* | *yes* |  |
| --- | --- | --- | --- | --- | --- | --- | --- | --- | --- | --- |
|  |  | *Multiple comparisons adjustment:* | | *n/adj* | *n/adj* | *Bonfer* | *Bonfer* | *Sidak* | *Scheffe* |  |
| **Comparison Superclass** |  | **Base Superclass** | **Difference** | **p-value of t-test** | | | | | | **Conclusion** |
| arts & culture | vs | africa | **-0.06701** | *0.0000* | *0.0000* | *0.0000* | *0.0000* | *0.0000* | *0.0000* | **diff is signif** |
| asia | vs | africa | **0.00042** | *0.9080* | *0.9120* | *1.0000* | *1.0000* | *1.0000* | *1.0000* |  |
| business & economy | vs | africa | **-0.04193** | *0.0000* | *0.0000* | *0.0000* | *0.0000* | *0.0000* | *0.0000* | **diff is signif** |
| envir & weather | vs | africa | **-0.06884** | *0.0000* | *0.0000* | *0.0000* | *0.0000* | *0.0000* | *0.0000* | **diff is signif** |
| europe | vs | africa | **0.00843** | *0.0130* | *0.0150* | *1.0000* | *1.0000* | *0.9280* | *0.9970* |  |
| health | vs | africa | **-0.06019** | *0.0000* | *0.0000* | *0.0020* | *0.0090* | *0.0090* | *0.5630* | **diff is signif** |
| human rights | vs | africa | **0.02441** | *0.0130* | *0.0090* | *1.0000* | *1.0000* | *0.7950* | *0.9920* |  |
| israel-palestine | vs | africa | **0.10397** | *0.0000* | *0.0000* | *0.0000* | *0.0000* | *0.0000* | *0.0000* | **diff is signif** |
| latin america | vs | africa | **-0.01131** | *0.0870* | *0.1300* | *1.0000* | *1.0000* | *1.0000* | *1.0000* |  |
| media | vs | africa | **0.00395** | *0.2940* | *0.3190* | *1.0000* | *1.0000* | *1.0000* | *1.0000* |  |
| middle east | vs | africa | **0.04613** | *0.0000* | *0.0000* | *0.0000* | *0.0000* | *0.0000* | *0.0000* | **diff is signif** |
| politics | vs | africa | **-0.00001** | *0.9980* | *0.9980* | *1.0000* | *1.0000* | *1.0000* | *1.0000* |  |
| racism | vs | africa | **0.11409** | *0.0000* | *0.0000* | *0.0000* | *0.0000* | *0.0000* | *0.0060* | **diff is signif** |
| russia | vs | africa | **0.05584** | *0.0000* | *0.0000* | *0.0050* | *0.0050* | *0.0050* | *0.4790* | **diff is signif** |
| science & technology | vs | africa | **-0.09338** | *0.0000* | *0.0000* | *0.0000* | *0.0000* | *0.0000* | *0.0000* | **diff is signif** |
| sport | vs | africa | **-0.05671** | *0.0060* | *0.0410* | *1.0000* | *1.0000* | *0.9990* | *1.0000* |  |
| us & canada | vs | africa | **0.00573** | *0.1670* | *0.1500* | *1.0000* | *1.0000* | *1.0000* | *1.0000* |  |
| war & conflict | vs | africa | **0.05278** | *0.0000* | *0.0000* | *0.0000* | *0.0000* | *0.0000* | *0.0000* | **diff is signif** |
| asia | vs | arts & culture | **0.06744** | *0.0000* | *0.0000* | *0.0000* | *0.0000* | *0.0000* | *0.0000* | **diff is signif** |
| business & economy | vs | arts & culture | **0.02508** | *0.0100* | *0.0130* | *1.0000* | *1.0000* | *0.8950* | *0.9960* |  |
| envir & weather | vs | arts & culture | **-0.00183** | *0.8810* | *0.8850* | *1.0000* | *1.0000* | *1.0000* | *1.0000* |  |
| europe | vs | arts & culture | **0.07544** | *0.0000* | *0.0000* | *0.0000* | *0.0000* | *0.0000* | *0.0000* | **diff is signif** |
| health | vs | arts & culture | **0.00682** | *0.6660* | *0.6870* | *1.0000* | *1.0000* | *1.0000* | *1.0000* |  |
| human rights | vs | arts & culture | **0.09143** | *0.0000* | *0.0000* | *0.0000* | *0.0000* | *0.0000* | *0.0000* | **diff is signif** |
| israel-palestine | vs | arts & culture | **0.17098** | *0.0000* | *0.0000* | *0.0000* | *0.0000* | *0.0000* | *0.0000* | **diff is signif** |
| latin america | vs | arts & culture | **0.05571** | *0.0000* | *0.0000* | *0.0000* | *0.0000* | *0.0000* | *0.1090* | **diff is signif** |
| media | vs | arts & culture | **0.07096** | *0.0000* | *0.0000* | *0.0000* | *0.0000* | *0.0000* | *0.0000* | **diff is signif** |
| middle east | vs | arts & culture | **0.11314** | *0.0000* | *0.0000* | *0.0000* | *0.0000* | *0.0000* | *0.0000* | **diff is signif** |
| politics | vs | arts & culture | **0.06700** | *0.0000* | *0.0000* | *0.0000* | *0.0000* | *0.0000* | *0.0000* | **diff is signif** |
| racism | vs | arts & culture | **0.18110** | *0.0000* | *0.0000* | *0.0000* | *0.0000* | *0.0000* | *0.0000* | **diff is signif** |
| russia | vs | arts & culture | **0.12286** | *0.0000* | *0.0000* | *0.0000* | *0.0000* | *0.0000* | *0.0000* | **diff is signif** |
| science & technology | vs | arts & culture | **-0.02637** | *0.0250* | *0.0220* | *1.0000* | *1.0000* | *0.9790* | *0.9980* |  |
| sport | vs | arts & culture | **0.01030** | *0.6390* | *0.7210* | *1.0000* | *1.0000* | *1.0000* | *1.0000* |  |
| us & canada | vs | arts & culture | **0.07274** | *0.0000* | *0.0000* | *0.0000* | *0.0000* | *0.0000* | *0.0000* | **diff is signif** |
| war & conflict | vs | arts & culture | **0.11979** | *0.0000* | *0.0000* | *0.0000* | *0.0000* | *0.0000* | *0.0000* | **diff is signif** |
| business & economy | vs | asia | **-0.04235** | *0.0000* | *0.0000* | *0.0000* | *0.0000* | *0.0000* | *0.0000* | **diff is signif** |
| envir & weather | vs | asia | **-0.06926** | *0.0000* | *0.0000* | *0.0000* | *0.0000* | *0.0000* | *0.0000* | **diff is signif** |
| europe | vs | asia | **0.00800** | *0.0320* | *0.0270* | *1.0000* | *1.0000* | *0.9910* | *0.9990* |  |
| health | vs | asia | **-0.06061** | *0.0000* | *0.0000* | *0.0020* | *0.0080* | *0.0080* | *0.5520* | **diff is signif** |
| human rights | vs | asia | **0.02399** | *0.0160* | *0.0110* | *1.0000* | *1.0000* | *0.8490* | *0.9940* |  |
| israel-palestine | vs | asia | **0.10354** | *0.0000* | *0.0000* | *0.0000* | *0.0000* | *0.0000* | *0.0000* | **diff is signif** |
| latin america | vs | asia | **-0.01173** | *0.0840* | *0.1190* | *1.0000* | *1.0000* | *1.0000* | *1.0000* |  |
| media | vs | asia | **0.00352** | *0.3870* | *0.3890* | *1.0000* | *1.0000* | *1.0000* | *1.0000* |  |
| middle east | vs | asia | **0.04570** | *0.0000* | *0.0000* | *0.0000* | *0.0000* | *0.0000* | *0.0000* | **diff is signif** |
| politics | vs | asia | **-0.00044** | *0.9320* | *0.9330* | *1.0000* | *1.0000* | *1.0000* | *1.0000* |  |
| racism | vs | asia | **0.11366** | *0.0000* | *0.0000* | *0.0000* | *0.0000* | *0.0000* | *0.0070* | **diff is signif** |
| russia | vs | asia | **0.05542** | *0.0000* | *0.0000* | *0.0060* | *0.0060* | *0.0050* | *0.5040* | **diff is signif** |
| science & technology | vs | asia | **-0.09380** | *0.0000* | *0.0000* | *0.0000* | *0.0000* | *0.0000* | *0.0000* | **diff is signif** |
| sport | vs | asia | **-0.05714** | *0.0060* | *0.0390* | *0.9780* | *1.0000* | *0.9990* | *1.0000* |  |
| us & canada | vs | asia | **0.00530** | *0.2310* | *0.1970* | *1.0000* | *1.0000* | *1.0000* | *1.0000* |  |
| war & conflict | vs | asia | **0.05236** | *0.0000* | *0.0000* | *0.0000* | *0.0000* | *0.0000* | *0.0000* | **diff is signif** |
| envir & weather | vs | business & economy | **-0.02691** | *0.0130* | *0.0130* | *1.0000* | *1.0000* | *0.8940* | *0.9950* |  |
| europe | vs | business & economy | **0.05036** | *0.0000* | *0.0000* | *0.0000* | *0.0000* | *0.0000* | *0.0000* | **diff is signif** |
| health | vs | business & economy | **-0.01826** | *0.2160* | *0.2420* | *1.0000* | *1.0000* | *1.0000* | *1.0000* |  |
| human rights | vs | business & economy | **0.06634** | *0.0000* | *0.0000* | *0.0000* | *0.0000* | *0.0000* | *0.0020* | **diff is signif** |
| israel-palestine | vs | business & economy | **0.14589** | *0.0000* | *0.0000* | *0.0000* | *0.0000* | *0.0000* | *0.0000* | **diff is signif** |
| latin america | vs | business & economy | **0.03062** | *0.0000* | *0.0010* | *0.0420* | *0.0960* | *0.0920* | *0.8520* |  |
| media | vs | business & economy | **0.04588** | *0.0000* | *0.0000* | *0.0000* | *0.0000* | *0.0000* | *0.0000* | **diff is signif** |
| middle east | vs | business & economy | **0.08805** | *0.0000* | *0.0000* | *0.0000* | *0.0000* | *0.0000* | *0.0000* | **diff is signif** |
| politics | vs | business & economy | **0.04191** | *0.0000* | *0.0000* | *0.0000* | *0.0000* | *0.0000* | *0.0070* | **diff is signif** |
| racism | vs | business & economy | **0.15601** | *0.0000* | *0.0000* | *0.0000* | *0.0000* | *0.0000* | *0.0000* | **diff is signif** |
| russia | vs | business & economy | **0.09777** | *0.0000* | *0.0000* | *0.0000* | *0.0000* | *0.0000* | *0.0000* | **diff is signif** |
| science & technology | vs | business & economy | **-0.05145** | *0.0000* | *0.0000* | *0.0000* | *0.0000* | *0.0000* | *0.0470* | **diff is signif** |
| sport | vs | business & economy | **-0.01479** | *0.4860* | *0.5990* | *1.0000* | *1.0000* | *1.0000* | *1.0000* |  |
| us & canada | vs | business & economy | **0.04766** | *0.0000* | *0.0000* | *0.0000* | *0.0000* | *0.0000* | *0.0000* | **diff is signif** |
| war & conflict | vs | business & economy | **0.09471** | *0.0000* | *0.0000* | *0.0000* | *0.0000* | *0.0000* | *0.0000* | **diff is signif** |
| europe | vs | envir& weather | **0.07727** | *0.0000* | *0.0000* | *0.0000* | *0.0000* | *0.0000* | *0.0000* | **diff is signif** |
| health | vs | envir& weather | **0.00865** | *0.6000* | *0.6180* | *1.0000* | *1.0000* | *1.0000* | *1.0000* |  |
| human rights | vs | envir& weather | **0.09325** | *0.0000* | *0.0000* | *0.0000* | *0.0000* | *0.0000* | *0.0000* | **diff is signif** |
| israel-palestine | vs | envir& weather | **0.17280** | *0.0000* | *0.0000* | *0.0000* | *0.0000* | *0.0000* | *0.0000* | **diff is signif** |
| latin america | vs | envir& weather | **0.05753** | *0.0000* | *0.0000* | *0.0000* | *0.0000* | *0.0000* | *0.1460* | **diff is signif** |
| media | vs | envir& weather | **0.07279** | *0.0000* | *0.0000* | *0.0000* | *0.0000* | *0.0000* | *0.0000* | **diff is signif** |
| middle east | vs | envir& weather | **0.11496** | *0.0000* | *0.0000* | *0.0000* | *0.0000* | *0.0000* | *0.0000* | **diff is signif** |
| politics | vs | envir& weather | **0.06883** | *0.0000* | *0.0000* | *0.0000* | *0.0000* | *0.0000* | *0.0000* | **diff is signif** |
| racism | vs | envir& weather | **0.18292** | *0.0000* | *0.0000* | *0.0000* | *0.0000* | *0.0000* | *0.0000* | **diff is signif** |
| russia | vs | envir& weather | **0.12468** | *0.0000* | *0.0000* | *0.0000* | *0.0000* | *0.0000* | *0.0000* | **diff is signif** |
| science & technology | vs | envir& weather | **-0.02454** | *0.0520* | *0.0440* | *1.0000* | *1.0000* | *1.0000* | *1.0000* |  |
| sport | vs | envir& weather | **0.01213** | *0.5900* | *0.6770* | *1.0000* | *1.0000* | *1.0000* | *1.0000* |  |
| us & canada | vs | envir& weather | **0.07457** | *0.0000* | *0.0000* | *0.0000* | *0.0000* | *0.0000* | *0.0000* | **diff is signif** |
| war & conflict | vs | envir& weather | **0.12162** | *0.0000* | *0.0000* | *0.0000* | *0.0000* | *0.0000* | *0.0000* | **diff is signif** |
| health | vs | europe | **-0.06862** | *0.0000* | *0.0000* | *0.0000* | *0.0010* | *0.0010* | *0.2550* | **diff is signif** |
| human rights | vs | europe | **0.01599** | *0.1070* | *0.0850* | *1.0000* | *1.0000* | *1.0000* | *1.0000* |  |
| israel-palestine | vs | europe | **0.09554** | *0.0000* | *0.0000* | *0.0000* | *0.0000* | *0.0000* | *0.0000* | **diff is signif** |
| latin america | vs | europe | **-0.01973** | *0.0030* | *0.0070* | *0.5080* | *1.0000* | *0.7140* | *0.9880* |  |
| media | vs | europe | **-0.00448** | *0.2430* | *0.2340* | *1.0000* | *1.0000* | *1.0000* | *1.0000* |  |
| middle east | vs | europe | **0.03770** | *0.0000* | *0.0000* | *0.0000* | *0.0000* | *0.0000* | *0.0000* | **diff is signif** |
| politics | vs | europe | **-0.00844** | *0.0850* | *0.0860* | *1.0000* | *1.0000* | *1.0000* | *1.0000* |  |
| racism | vs | europe | **0.10566** | *0.0000* | *0.0000* | *0.0000* | *0.0000* | *0.0000* | *0.0250* | **diff is signif** |
| russia | vs | europe | **0.04742** | *0.0000* | *0.0000* | *0.0670* | *0.0580* | *0.0560* | *0.8010* |  |
| science & technology | vs | europe | **-0.10181** | *0.0000* | *0.0000* | *0.0000* | *0.0000* | *0.0000* | *0.0000* | **diff is signif** |
| sport | vs | europe | **-0.06514** | *0.0020* | *0.0190* | *0.2720* | *1.0000* | *0.9600* | *0.9980* |  |
| us & canada | vs | europe | **-0.00270** | *0.5220* | *0.4760* | *1.0000* | *1.0000* | *1.0000* | *1.0000* |  |
| war & conflict | vs | europe | **0.04435** | *0.0000* | *0.0000* | *0.0000* | *0.0000* | *0.0000* | *0.0000* | **diff is signif** |
| human rights | vs | health | **0.08461** | *0.0000* | *0.0000* | *0.0000* | *0.0000* | *0.0000* | *0.1460* | **diff is signif** |
| israel-palestine | vs | health | **0.16416** | *0.0000* | *0.0000* | *0.0000* | *0.0000* | *0.0000* | *0.0000* | **diff is signif** |
| latin america | vs | health | **0.04889** | *0.0010* | *0.0030* | *0.1870* | *0.4370* | *0.3540* | *0.9570* |  |
| media | vs | health | **0.06414** | *0.0000* | *0.0000* | *0.0010* | *0.0030* | *0.0030* | *0.4250* | **diff is signif** |
| middle east | vs | health | **0.10632** | *0.0000* | *0.0000* | *0.0000* | *0.0000* | *0.0000* | *0.0000* | **diff is signif** |
| politics | vs | health | **0.06018** | *0.0000* | *0.0000* | *0.0040* | *0.0140* | *0.0140* | *0.6230* | **diff is signif** |
| racism | vs | health | **0.17428** | *0.0000* | *0.0000* | *0.0000* | *0.0000* | *0.0000* | *0.0000* | **diff is signif** |
| russia | vs | health | **0.11604** | *0.0000* | *0.0000* | *0.0000* | *0.0000* | *0.0000* | *0.0090* | **diff is signif** |
| science & technology | vs | health | **-0.03319** | *0.0400* | *0.0450* | *1.0000* | *1.0000* | *1.0000* | *1.0000* |  |
| sport | vs | health | **0.00348** | *0.8880* | *0.9110* | *1.0000* | *1.0000* | *1.0000* | *1.0000* |  |
| us & canada | vs | health | **0.06592** | *0.0000* | *0.0000* | *0.0000* | *0.0020* | *0.0020* | *0.3610* | **diff is signif** |
| war & conflict | vs | health | **0.11297** | *0.0000* | *0.0000* | *0.0000* | *0.0000* | *0.0000* | *0.0000* | **diff is signif** |
| israel-palestine | vs | human rights | **0.07955** | *0.0000* | *0.0000* | *0.0000* | *0.0000* | *0.0000* | *0.0000* | **diff is signif** |
| latin america | vs | human rights | **-0.03572** | *0.0020* | *0.0020* | *0.2980* | *0.2960* | *0.2560* | *0.9380* |  |
| media | vs | human rights | **-0.02047** | *0.0410* | *0.0310* | *1.0000* | *1.0000* | *0.9950* | *0.9990* |  |
| middle east | vs | human rights | **0.02171** | *0.0280* | *0.0190* | *1.0000* | *1.0000* | *0.9650* | *0.9980* |  |
| politics | vs | human rights | **-0.02443** | *0.0200* | *0.0150* | *1.0000* | *1.0000* | *0.9190* | *0.9960* |  |
| racism | vs | human rights | **0.08967** | *0.0000* | *0.0000* | *0.0160* | *0.0030* | *0.0030* | *0.4100* | **diff is signif** |
| russia | vs | human rights | **0.03143** | *0.0530* | *0.0470* | *1.0000* | *1.0000* | *1.0000* | *1.0000* |  |
| science & technology | vs | human rights | **-0.11779** | *0.0000* | *0.0000* | *0.0000* | *0.0000* | *0.0000* | *0.0000* | **diff is signif** |
| sport | vs | human rights | **-0.08113** | *0.0000* | *0.0050* | *0.0570* | *0.8850* | *0.5880* | *0.9810* |  |
| us & canada | vs | human rights | **-0.01869** | *0.0670* | *0.0490* | *1.0000* | *1.0000* | *1.0000* | *1.0000* |  |
| war & conflict | vs | human rights | **0.02837** | *0.0120* | *0.0080* | *1.0000* | *1.0000* | *0.7410* | *0.9900* |  |
| latin america | vs | israel-palestine | **-0.11527** | *0.0000* | *0.0000* | *0.0000* | *0.0000* | *0.0000* | *0.0000* | **diff is signif** |
| media | vs | israel-palestine | **-0.10002** | *0.0000* | *0.0000* | *0.0000* | *0.0000* | *0.0000* | *0.0000* | **diff is signif** |
| middle east | vs | israel-palestine | **-0.05784** | *0.0000* | *0.0000* | *0.0000* | *0.0000* | *0.0000* | *0.0000* | **diff is signif** |
| politics | vs | israel-palestine | **-0.10398** | *0.0000* | *0.0000* | *0.0000* | *0.0000* | *0.0000* | *0.0000* | **diff is signif** |
| racism | vs | israel-palestine | **0.01012** | *0.6370* | *0.5970* | *1.0000* | *1.0000* | *1.0000* | *1.0000* |  |
| russia | vs | israel-palestine | **-0.04812** | *0.0010* | *0.0000* | *0.1100* | *0.0740* | *0.0710* | *0.8270* |  |
| science & technology | vs | israel-palestine | **-0.19735** | *0.0000* | *0.0000* | *0.0000* | *0.0000* | *0.0000* | *0.0000* | **diff is signif** |
| sport | vs | israel-palestine | **-0.16068** | *0.0000* | *0.0000* | *0.0000* | *0.0000* | *0.0000* | *0.0160* | **diff is signif** |
| us & canada | vs | israel-palestine | **-0.09824** | *0.0000* | *0.0000* | *0.0000* | *0.0000* | *0.0000* | *0.0000* | **diff is signif** |
| war & conflict | vs | israel-palestine | **-0.05119** | *0.0000* | *0.0000* | *0.0000* | *0.0000* | *0.0000* | *0.0000* | **diff is signif** |
| media | vs | latin america | **0.01525** | *0.0260* | *0.0450* | *1.0000* | *1.0000* | *1.0000* | *1.0000* |  |
| middle east | vs | latin america | **0.05743** | *0.0000* | *0.0000* | *0.0000* | *0.0000* | *0.0000* | *0.0000* | **diff is signif** |
| politics | vs | latin america | **0.01129** | *0.1310* | *0.1700* | *1.0000* | *1.0000* | *1.0000* | *1.0000* |  |
| racism | vs | latin america | **0.12539** | *0.0000* | *0.0000* | *0.0000* | *0.0000* | *0.0000* | *0.0020* | **diff is signif** |
| russia | vs | latin america | **0.06715** | *0.0000* | *0.0000* | *0.0010* | *0.0010* | *0.0010* | *0.2990* | **diff is signif** |
| science & technology | vs | latin america | **-0.08207** | *0.0000* | *0.0000* | *0.0000* | *0.0000* | *0.0000* | *0.0000* | **diff is signif** |
| sport | vs | latin america | **-0.04541** | *0.0340* | *0.1110* | *1.0000* | *1.0000* | *1.0000* | *1.0000* |  |
| us & canada | vs | latin america | **0.01703** | *0.0160* | *0.0250* | *1.0000* | *1.0000* | *0.9870* | *0.9990* |  |
| war & conflict | vs | latin america | **0.06409** | *0.0000* | *0.0000* | *0.0000* | *0.0000* | *0.0000* | *0.0000* | **diff is signif** |
| middle east | vs | media | **0.04218** | *0.0000* | *0.0000* | *0.0000* | *0.0000* | *0.0000* | *0.0000* | **diff is signif** |
| politics | vs | media | **-0.00396** | *0.4420* | *0.4530* | *1.0000* | *1.0000* | *1.0000* | *1.0000* |  |
| racism | vs | media | **0.11014** | *0.0000* | *0.0000* | *0.0000* | *0.0000* | *0.0000* | *0.0130* | **diff is signif** |
| russia | vs | media | **0.05190** | *0.0000* | *0.0000* | *0.0200* | *0.0180* | *0.0180* | *0.6570* | **diff is signif** |
| science & technology | vs | media | **-0.09733** | *0.0000* | *0.0000* | *0.0000* | *0.0000* | *0.0000* | *0.0000* | **diff is signif** |
| sport | vs | media | **-0.06066** | *0.0030* | *0.0290* | *0.5770* | *1.0000* | *0.9930* | *0.9990* |  |
| us & canada | vs | media | **0.00178** | *0.6930* | *0.6750* | *1.0000* | *1.0000* | *1.0000* | *1.0000* |  |
| war & conflict | vs | media | **0.04883** | *0.0000* | *0.0000* | *0.0000* | *0.0000* | *0.0000* | *0.0000* | **diff is signif** |
| politics | vs | middle east | **-0.04614** | *0.0000* | *0.0000* | *0.0000* | *0.0000* | *0.0000* | *0.0000* | **diff is signif** |
| racism | vs | middle east | **0.06796** | *0.0010* | *0.0000* | *0.2010* | *0.0520* | *0.0510* | *0.7890* |  |
| russia | vs | middle east | **0.00972** | *0.4660* | *0.4630* | *1.0000* | *1.0000* | *1.0000* | *1.0000* |  |
| science & technology | vs | middle east | **-0.13951** | *0.0000* | *0.0000* | *0.0000* | *0.0000* | *0.0000* | *0.0000* | **diff is signif** |
| sport | vs | middle east | **-0.10284** | *0.0000* | *0.0000* | *0.0000* | *0.0350* | *0.0340* | *0.7420* | **diff is signif** |
| us & canada | vs | middle east | **-0.04040** | *0.0000* | *0.0000* | *0.0000* | *0.0000* | *0.0000* | *0.0000* | **diff is signif** |
| war & conflict | vs | middle east | **0.00666** | *0.2970* | *0.2800* | *1.0000* | *1.0000* | *1.0000* | *1.0000* |  |
| racism | vs | politics | **0.11410** | *0.0000* | *0.0000* | *0.0000* | *0.0000* | *0.0000* | *0.0080* | **diff is signif** |
| russia | vs | politics | **0.05586** | *0.0000* | *0.0000* | *0.0090* | *0.0080* | *0.0080* | *0.5560* | **diff is signif** |
| science & technology | vs | politics | **-0.09337** | *0.0000* | *0.0000* | *0.0000* | *0.0000* | *0.0000* | *0.0000* | **diff is signif** |
| sport | vs | politics | **-0.05670** | *0.0070* | *0.0420* | *1.0000* | *1.0000* | *0.9990* | *1.0000* |  |
| us & canada | vs | politics | **0.00574** | *0.2920* | *0.2780* | *1.0000* | *1.0000* | *1.0000* | *1.0000* |  |
| war & conflict | vs | politics | **0.05279** | *0.0000* | *0.0000* | *0.0000* | *0.0000* | *0.0000* | *0.0000* | **diff is signif** |
| russia | vs | racism | **-0.05824** | *0.0180* | *0.0110* | *1.0000* | *1.0000* | *0.8370* | *0.9930* |  |
| science & technology | vs | racism | **-0.20747** | *0.0000* | *0.0000* | *0.0000* | *0.0000* | *0.0000* | *0.0000* | **diff is signif** |
| sport | vs | racism | **-0.17080** | *0.0000* | *0.0000* | *0.0000* | *0.0000* | *0.0000* | *0.0930* | **diff is signif** |
| us & canada | vs | racism | **-0.10836** | *0.0000* | *0.0000* | *0.0000* | *0.0000* | *0.0000* | *0.0180* | **diff is signif** |
| war & conflict | vs | racism | **-0.06131** | *0.0050* | *0.0020* | *0.7940* | *0.2910* | *0.2520* | *0.9370* |  |
| science & technology | vs | russia | **-0.14922** | *0.0000* | *0.0000* | *0.0000* | *0.0000* | *0.0000* | *0.0000* | **diff is signif** |
| sport | vs | russia | **-0.11256** | *0.0000* | *0.0000* | *0.0010* | *0.0380* | *0.0380* | *0.7540* | **diff is signif** |
| us & canada | vs | russia | **-0.05012** | *0.0000* | *0.0000* | *0.0380* | *0.0310* | *0.0300* | *0.7270* | **diff is signif** |
| war & conflict | vs | russia | **-0.00306** | *0.8320* | *0.8300* | *1.0000* | *1.0000* | *1.0000* | *1.0000* |  |
| sport | vs | science & technology | **0.03667** | *0.0990* | *0.2010* | *1.0000* | *1.0000* | *1.0000* | *1.0000* |  |
| us & canada | vs | science & technology | **0.09911** | *0.0000* | *0.0000* | *0.0000* | *0.0000* | *0.0000* | *0.0000* | **diff is signif** |
| war & conflict | vs | science & technology | **0.14616** | *0.0000* | *0.0000* | *0.0000* | *0.0000* | *0.0000* | *0.0000* | **diff is signif** |
| us & canada | vs | sport | **0.06244** | *0.0030* | *0.0240* | *0.4520* | *1.0000* | *0.9850* | *0.9990* |  |
| war & conflict | vs | sport | **0.10949** | *0.0000* | *0.0000* | *0.0000* | *0.0170* | *0.0170* | *0.6550* | **diff is signif** |
| war & conflict | vs | us & canada | **0.04705** | *0.0000* | *0.0000* | *0.0000* | *0.0000* | *0.0000* | *0.0000* | **diff is signif** |
